# Vaccination strategies on dynamic networks with indirect transmission links and limited contact information

**DOI:** 10.1371/journal.pone.0241612

**Published:** 2020-11-12

**Authors:** Md Shahzamal, Bernard Mans, Frank de Hoog, Dean Paini, Raja Jurdak

**Affiliations:** 1 Data61, CSIRO, Brisbane, Australia; 2 Macquarie University, Sydney, Australia; 3 Data61, CSIRO, Canberra, Australia; 4 Health and Biosecurity, CSIRO, Canberra, Australia; 5 Queensland University of Technology, Brisbane, Australia; University of Waterloo, CANADA

## Abstract

Infectious diseases are still a major global burden for modern society causing 13 million deaths annually. One way to reduce the morbidity and mortality rates from infectious diseases is through pre-emptive or targeted vaccinations. Current theoretical vaccination strategies based on contact networks, however, rely on highly specific individual contact information which is difficult and costly to obtain, in order to identify influential spreading individuals. Current approaches also focus only on direct contacts between individuals for spreading, and disregard indirect transmission where a pathogen can spread between one infected individual and one susceptible individual who visit the same location within a short time-frame without meeting. This paper presents a novel vaccination strategy which relies on coarse-grained contact information, both direct and indirect, that can be easily and efficiently collected. Rather than tracking exact contact degrees of individuals, our strategy uses the types of places people visit to estimate a range of contact degrees for individuals, considering both direct and indirect contacts. We conduct extensive computer simulations to evaluate the performance of our strategy in comparison to state-of-the-art vaccination strategies. Results show that, when considering indirect links, our lower cost vaccination strategy achieves comparable performance to the contact-degree based approach and outperforms other existing strategies without requiring over-detailed information.

## Introduction

In addition to basic health and survival concerns, infectious diseases also represent a global burden for modern society due to their substantial economic and social impacts, as is demonstrated by the current COVID-19 outbreak. Various theoretical strategies have been devised to reduce the number of casualties and overall impact. One strategy is to vaccinate the population before an outbreak occurs. This vaccination is called pre-emptive vaccination as it is applied to prevent future outbreaks of the known infectious diseases. However, if there is a new strain of a virus, reactive vaccinations (once generated) can be implemented to hinder the spread of a new disease for an ongoing outbreak. There have been several theoretical studies of well-documented pre-emptive and reactive vaccinations strategies to prevent spreading of infectious diseases [1–3]. The development of current theoretical vaccination strategies can be divided into two steps: i) studying various vaccination strategies in a virtual environment by simulating the disease spread on social contact networks with vaccination strategies; and ii) using the results of the simulations to inform the actual strategy [4–6].

Existing vaccination strategies aim to identify a set of individuals who are influential disease spreaders for vaccination to reduce the rate at which the infectious disease can spread [7–9]. An essential task for implementing a vaccination strategy, therefore, is to identify an appropriate set of individuals and vaccinate them to modify the spreading dynamics. Therefore, the goals of a theoretical study are that the size of the selected set of individuals should be minimal and should only require realistic resources (taking into account vaccination cost, information collection cost and manpower) to achieve the required prevention.

Previous research has found that infection transmission from an infected individual to a susceptible individual depends on the movement and interaction patterns of both susceptible and infected individuals along with other biological factors [10]. Thus, interaction patterns are often analysed to find appropriate vaccination strategies [10, 11]. Applying detailed information about individual interactions to develop a strategy will often result in effective vaccination. However, higher order interaction information (e.g. contact duration, time of contact, and whom an individual interacted at each contact etc.) is quite difficult to obtain for real-world social contact networks because of the collection complexity and privacy issues. Computing diffusion control parameters such as betweenness centrality, eigenvalue and closeness [8, 9, 12] are often impractical for vaccination purposes and thus vaccination strategies are often solely developed based on locally obtainable contact information, i.e. contact information that an individual can readily remember and report such as where they have visited, how many people they have met and how long they have stayed in the visited location etc. This paper, therefore, focuses on studying vaccination strategies using local contact information.

There has been a wide range of methods to reduce the spread of disease on contact networks using local contact information. The simplest one is random vaccination (RV) where a proportion of a population is randomly chosen to be vaccinated [3, 13, 14]. Unfortunately, this strategy requires a large number of individuals to be vaccinated. Another simple vaccination approach is the acquaintance vaccination (AV) where a random individual is approached and asked to name a friend [3, 13]. A name recommended by multiple individuals increases the preference to be vaccinated [3]. This strategy avoids the random selection of RV strategy and provides an opportunity to select individuals who have contact with many other individuals. It is a targeted vaccination where the most influential individuals are removed from the disease transmission path. Although the AV approach would perform much better than the vaccination strategies that is being currently used in the real world, it would still require a large number of individuals to be vaccinated. There have been several modifications to the AV strategy in order to make it more efficient [15]. However, these strategies are only studied on static contact networks. The work of [16] has upgraded the AV strategy by prioritising the most recently contacted neighbours, encountered individuals or nodes at location, and assigning weights to their links to capture contact frequency. These vaccination strategies use the recommendation of a neighbouring individual from another individual. However, such information is often inaccurate and hence an optimal set of individuals may not be selected for vaccination.

Another limitation of current contact-based vaccination strategies is that they only focus on direct contacts between individuals. Our recent work [17–20] introduced the concept of indirect transmission, where disease can transmit through indirect interaction (in addition to direct interactions), which is representative of many infectious diseases. For example, a susceptible individual may get infected by visiting the locations where an airborne disease infected individual has been even after that individual has left the location. This is because infectious particles generated by the infected individual persist in the environment, which can transfer to the visiting susceptible individuals. Indirect transmissions may occur via a *fomite* (e.g., an object like a doorknob or paper that has been contaminated with infectious virus) [21] or even by the eventual inhalation of droplet nuclei in the air forming *aerosolized fomites* (such as microscopic aerosol particles consisting of the residual solid cores of evaporated respiratory droplets) [22].

Current vaccination strategies are not designed to capture indirect transmissions, thus potentially missing highly influential individuals with many indirect links. This work investigates the development of appropriate vaccination strategies on dynamic contact networks capturing indirect transmission links as well as direct transmission links.

In particular, this paper proposes a local contact information based strategy, called the *individual’s movement based vaccination (IMV)* strategy, where individuals are vaccinated based on their movement behaviours. It is common for individuals to not provide accurate contact information when asked about their past interactions. Instead, they give a rough estimation of their contact information. In the proposed strategy, individuals are ranked based on their movements relative to public places. Individuals are asked about the frequencies of visits to the different classes of locations (classified based on the intensity of individual visits to those locations, such as the temporal popularity of the places). The locations are labelled with a range of values indicating the number of individuals that could be met when the location is visited by an individual. Based on this coarse-grained information, ranking scores are estimated for the sample individuals (picked up as candidates for the vaccine) from a population, and the highest ranked individuals are vaccinated. This approach can be suitable as it is easier to gather individual movement information than the contacts they create with others. Secondly, individuals can easily remember the visited locations (such as bars) rather than how many people and whom they have met. The performance of the proposed vaccination strategy is analysed for the spread of airborne infectious disease. The susceptible-infectious-recovered (SIR) epidemic model is applied to simulate the disease spreading on dynamic contact networks. First, the final outbreak sizes are obtained without vaccination, then the effectiveness of the proposed strategy is analysed by estimating the reduction in the outbreak sizes compared to those without vaccination. The efficacy of the proposed strategy is compared with three other strategies: random vaccination (RV), acquaintance vaccination (AV), and degree-based vaccination (DV) where higher degree individuals are vaccinated [23].

Experiments were conducted to study vaccination strategies for both pre-emptive and reactive vaccination scenarios. As individuals can only mention their direct interactions, vaccination strategies are first investigated by ranking nodes based on direct interactions. Then, it is determined how the vaccination strategies are affected if both direct and indirect interactions are accounted for selecting the nodes to be vaccinated. As the proposed strategy depends on coarse-grained information, the vaccination performance can deviate from that of the exact contact information based vaccination. Thus, the vaccination strategies are analysed by using the exact contact information. The proposed strategy is designed for dynamic contact networks even though the temporal information is not integrated with the node selection process. An investigation is done to understand the impact of integrating temporal information with the proposed strategy.

In the network based targeted vaccination campaign, the authorities should first collect information about the contact network and then can use this to vaccinate. Therefore, this process will involve three types of costs: the cost of the disease itself—outbreak sizes, the cost of collecting contact information—information availability, and the cost of vaccination—vaccination coverage. This means that the vaccination strategy should be efficient in terms of information collection cost. Thus, the other important focus of this paper is to examine the effectiveness of vaccination strategies with respect to the scale of information collection on node contacts [24]. The aims of this paper are as follows:

- Investigating the impact of indirect interactions on the performance of the current vaccination strategies,
- Developing a new vaccination strategy using the local approximate contact information and movement behaviours,
- Analysing the performance of the developed strategy in both pre-emptive and reactive scenarios and exploring the significance of the other contact information,
- Investigating how the scale of information availability influences the performance of the vaccination strategies and understanding what is the minimum required threshold to make a vaccination strategy efficient.

## Method and materials

This section describes the proposed vaccination strategy and the methods for analysing its effectiveness to reduce disease spreading.

### Proposed vaccination strategy

The proposed vaccination strategy, called the individual’s movement based vaccination (IMV) strategy, is based on the individual’s movement behaviours and propensity to interact with each other. The importance of an individual’s past movement behaviours and interaction propensity is reflected by an individual ranking score. The ranking scores are used as indicators of individual’s influence in spreading the disease in the near future. In the IMV strategy, locations where individuals visit daily such as offices, restaurants, shopping malls and schools etc. are classified based on the number of neighbour individuals an individual can meet if they visit these locations. The locations are intuitively grouped into six classes as in Table 1. Note that this classification is approximate and its accuracy is correlated to the effectiveness of the proposed vaccination strategy. In this strategy, individuals are asked the number of times they have visited the different classes of locations within a previous time period. The class range is used as individuals may not remember nor notice the exact number of the individuals they have contacted in their past visits to different locations. Instead, they give a rough estimate of their contacts. Individuals also forget to mention any short visits. Finally, it is easier to gather movement information of individuals instead of collecting information for every single visit.

**Table 1:** Classification of visits nodes do during their daily activities.

class	contact sizes	locations
class-1	1-5	home, store
class-2	6-15	coffee shop, bus stop
class-3	16-25	office, local train station, small park, swimming pools
class-4	26-50	central train station, large park, clubs and night clubs
class-5	51-100	shopping mall, college, central train station, sport centers
class-6	101-	university, college, airport, concerts, festival and football stadiums

A generic method is now developed to assess the rank of an individual based on the relevant movement information. Assuming that a susceptible individual *v* who has been at a location where an infected individual *u* has visited gets infected with probability *β*. The probability of *v* for not being infected due to this visit is 1 − *β*. If an infected individual *u* meets *d* individuals during this visit, the probability of transmitting disease to the neighbours through this visit is given by

$$\begin{array}{c} w=1-{\left(1-\beta \right)}^{d} \end{array}$$

where (1 − *β*)^*d*^ is the probability that no neighbour individual is infected from this visit [25]. Here, the assumption is that the individual *u* is only the source of infection. All the neighbouring individuals are susceptible and in contact with *u* independently. Under these assumptions, let *w* be the spreading potential for a visit by an infected individual at a location with a number of individuals. The spreading potential for visiting a location belonging to a class *i* can be approximated as

$$\begin{array}{c} w_{i}=\frac{1}{2}(2-{\left(1-\beta \right)}^{d_{i}^{1}}-{\left(1-\beta \right)}^{d_{i}^{2}}) \end{array}$$

where $d_{i}^{1}$ is the lower limit of class *i* and $d_{i}^{2}$ is the upper limit of class *i*. In fact, this is the average spreading potential for the class *i* locations. Then, the ranking score of an individual for visits to different classes of locations is given as

$$\begin{array}{c} W=\sum_{1}^{6}f_{i}\times w_{i} \end{array}$$ (1)

where *f*_*i*_ is the frequency of visit to a location belong to class *i*.

In this method, *W* can be interpreted as the maximum number of disease transmission events during the observation period. As this score is a relative value, it will carry significant information even if the same neighbouring individuals are met repeatedly. This is because repeated interaction increases the disease transmission opportunity. In addition, *W* indicates how easily a susceptible individual *v* gets infected due to movement behaviours and the propensity of interactions. Like the degree-based strategy (DV), the IMV strategy accounts for super-spreaders, but also accounts for the intensity of interactions among individuals through *d*_*i*_ and *f*_*i*_. It also accounts for the importance of places that DV strategy does not consider. In practice, it is also easier to remember the visited locations than how many people one has met.

### Experimental setup

The proposed vaccination strategy is analysed through simulating infectious disease spreading on the dynamic contact networks. We have used two types of contact networks: 1) a real contact network constructed from the GPS locations collected from users of a location based social discovery network Momo (www.immomo.com) [26]; and 2) a synthetic contact network generated by the same place different time (SPDT) graph model [17]. Both networks consider indirect transmission links along with direct links. Simulations on both networks allow us to understand the consistency of the results. We have also run simulations for other popular vaccination strategies to compare the performance. Here, we first describe the construction of the contact networks followed by disease propagation model, selected baseline vaccination strategies and performance metrics.

#### Data set and contact networks

This study applies movement information collected from Momo users. The Momo App enables users to interact with nearby users by sharing their current locations. Whenever a user launches the Momo app, the current location is forwarded to the Momo server. The server sends back the latest location updates of all users nearby. These location updates have been previously collected by the authors of [26] using a set of network API communicating with the Momo server. The API retrieved location updates every 15 minutes over a period of 71 days (from May to October 2012). The data set contains 356 million location updates from about 6 million Momo users around the world, but primarily in China. Each database entry includes GPS coordinates of the location, time of update and user ID. For this study, the updates from Beijing are extracted as it is the city with the highest number of updates for the period of 32 days from 17 September, 2012 to 19 October, 2012. This data contains almost 56 million location updates from 0.6 million users. Note that the data from MOMO has been provided under strict access rules. In addition, the data is anonymised and thus private information (e.g., age, gender, location type,…) cannot be provided.

All possible disease transmission links are extracted from this data set according to the SPDT diffusion model and a SPDT contact network of 338K users (see Section A.1 in Appendix-A). This network includes possible direct and indirect transmission links due to direct and indirect co-location interactions among users. However, users appear in the system for 3-4 days on average and then disappear for the remainder of the simulation period. Thus, the link density in the network is sparse. In this sparse SPDT network, infected individuals have limited potential to infect other individuals due to their short presence in the network which underestimates the diffusion dynamics. Thus, we reconstruct a dense SPDT network (DDT) from this network repeating the links from the available days of a user to the missing days for that user [27, 28]. If a user has links for multiple days, a day will be randomly chosen and will be copied to a randomly chosen day where that user has no links. Then, this 32 days contact network is extended to 42 days (6 weeks). In this extension, all links of a randomly selected day are copied to a day within the 32th to the 42th day period. Thus, a DDT network for 42 days with 338K users is obtained.

A synthetic SPDT contact network (GDT) is generated for 42 days with 368K nodes using the SPDT graph model introduced in our paper. In the GDT network, a node is active for a period of time which mimics a visit to a location where other nodes are present and the disease can be transmitted. The length of active periods is drawn from a geometric distribution. During the active period, a node contact with each neighbouring node is drawn from a power law distribution. The parameters of the power law distribution are heterogeneous as individuals have heterogeneous propensity to engage in contact with others. The delay a node takes before joining a host node and the duration the node stays with the host node are also drawn from the geometry distributions. Thus, the GDT network can simulate disease spreading with the proposed vaccination strategy. The GDT network can verify the experimental results obtained from the DDT contact network. If indirect links from both DDT and GDT networks are excluded, they provide the contact networks with direct links only. Networks with the direct links are called DST and GST respectively. From these networks, the contact information of the first 7 days is applied for ranking the nodes to vaccinate and the rest of the contact information is applied to simulate a disease spread.

#### Disease propagation

To propagate the disease on the selected contact networks, we consider a generic Susceptible-Infected-Recovered (SIR) epidemic model. In this model, nodes remain in one of the three compartments, namely, Susceptible (S), Infectious (I) and Recovered (R). The probability of infection for causing disease for an interaction can be determined by the dose-response relationship defined as

$$\begin{array}{c} P_{I}=1-e^{-\sigma E} \end{array}$$ (2)

where *σ* is the infectiousness of the virus that causes infection [29] and *E* is the received exposure for being in contact of infected individuals(see Section A.2 in Appendix-A). This value depends on the disease types and even virus types. If the susceptible individual move to the infected compartment with the probability *P*_*I*_, it continues to produce infectious particles over its infectious period *τ* days until they enter the recovered state, where 1/*τ* is the rate of recovering from the disease. In this model, no event of birth, death or migration of individuals is considered.

The simulations are forwarded in our experiments with one day interval [27, 28]. We chose an initial set of seed nodes, based on the requirements of the experiment, to start simulations assuming that it will be capable of showing the full epidemic curve in the studied simulation duration of 35 days. During each day of disease simulation, the received SPDT links for each susceptible individual from infected individuals are separated and infection probabilities are calculated by Eq 6. The volume *V* of proximity in Eq 5 is fixed to 2,512m^3^ assuming that the distance, within which a susceptible individual can inhale the infectious particles from an infected individual, is 20m and the particles will be available up to the height of 2m [29, 30]. The other parameters are assigned as follows: infectious particle generation rate *g* = 0.304 PFU (plaque-forming unit)/s and pulmonary rate *q* = 7.5 liter/min [30–32] when an individuals get infected with an airborne disease. Infectious particles may require 7.5 min to 300 min to be removed from interaction areas after their generation. We assign $r=\frac{1}{60b}$ to Eq 5 where *b* is the particle removal time randomly chosen from [7.5-300] min given a median particle removal time *r*_*t*_. This indicates how frequently infectious particles will leave a location. The parameter *σ* is set to 0.33 as the median value of required exposures for influenza to induce disease in 50% susceptible individuals is 2.1 PFU [33]. Susceptible individuals stochastically switch to the infected states in the next day of simulation according to the Bernoulli process with the infection probability *P*_*I*_ (Eq 6). Individuals stay infected up to *τ* days randomly picked up from 3-5 days maintaining $\bar{\tau }=4$ days (except when other ranges are mentioned explicitly) [34].

#### Baseline vaccination strategies

The performance of the proposed vaccination strategy is compared to three vaccination strategies:

Random vaccination (RV): this is a simple way of vaccination where nodes are chosen randomly to be vaccinated [23, 35]. To implement this process in pre-emptive vaccination scenarios, a percentage *P* of nodes are chosen randomly without knowing their contact behaviours and are vaccinated. The RV strategy is also applied in reactive scenarios where a percentage *P* of neighbouring nodes (whom the host node has contacted) of an infected node are chosen for vaccination. This approach for reactive scenarios is called ring vaccination with random acquaintance selection and is widely used.

Acquaintance vaccination (AV): in this strategy, a node is randomly approached and asked to name a neighbouring node to be vaccinated [13, 36]. To rank the nodes in this strategy, each node present in the network during the first seven days is asked to name a neighbour node and then a list is prepared with each recommended name (the same name can be listed multiple times as they can be recommended by multiple neighbouring nodes). Then, the number of repetitions of each name is counted and ranking scores are obtained for all nodes in the network. The nodes who have contact with a large number of nodes may be named more frequently. Then, *PN*/100 nodes are chosen from the top-ranked nodes based on the naming score to vaccinate percentage *P* of nodes, where *N* is the number of total nodes in the selected networks. AV fails to capture indirect transmission events as individuals do not have visibility into indirect contacts they have had, these contacts are due to visits to the same place at different times.

Degree-based vaccination (DV): this vaccination strategy vaccinates the nodes that have the largest number of contacts (high degree nodes) as they are more prone to get infected and spread disease [23]. It limits the problem of the above strategies that require substantial resources to prevent the spread of a disease. The contact set sizes for the first seven days of nodes in both networks are separated and the nodes are ranked based on the contact set sizes during this time. Then, *PN*/100 nodes are chosen from the top-ranked node based on the contact set sizes to vaccinate percentage *P* of nodes where *P* is the vaccination coverage. However, this strategy requires the exact information of all contacts a node has. It is often hard in practice to count explicitly all other individuals an individual has met, and may be unfeasible in real-world implementations. However, it is of interest to understand how effective the proposed strategy is compared to DV strategy, as an oracle approach. As for AV, the DV approach does not consider indirect transmission links and suffers from the same limitations.

#### Vaccination scenarios

The vaccination strategies are studied in two set of scenarios. In the first scenario, we investigate pre-emptive vaccination where vaccination is implemented to prevent possible future outbreaks of disease. In the second scenario, we investigate vaccination strategies for reactive vaccination where vaccination is implemented to hinder spreading of on going outbreak of a disease.

Reactive vaccination can be implemented in two ways. The first way is similar to the mass vaccination and is called population-level vaccination where a percentage *P* of the population is randomly chosen and is vaccinated. In the simplest version, behaviours of the selected individuals are not considered, randomly picked up to vaccinate, and hence information collection cost is minimal. However, the resource cost is often too high as it requires to vaccinate a large number of individuals. This approach is improved by vaccinating individuals who have specific characteristics relevant to disease spreading. For example, if an individual has interactions with many other individuals, he is chosen to be vaccinated (degree-based vaccination). In this method, the applied three ranking methods (IMV, DV and AV) of nodes are used to select the nodes to be vaccinated. In addition, random vaccination (RV) method is also examined along with these three ranking based methods.

In the second way of reactive vaccination implementation, the infected individuals are at the focus point where susceptible individuals who have contact with infected individuals are vaccinated to hinder further spreading of disease. This approach of implementing reactive vaccination is named as node level vaccination. In the node level vaccination, a percentage *P* of neighbour nodes are vaccinated and the neighbour nodes can be chosen randomly or based on a specific criterion. The two ranking methods (IMV and DV) are applied to select neighbours in the ring vaccination and the performance of node level vaccination is studied. The performance is compared with the strategy of random neighbour selection (RV).

The vaccination strategies are also examined with the scale *F* of information availability on the node’s contact. For node level vaccination, all infected node might not be identified. Thus, the performance is analysed only if a proportion *F* of infected nodes is identified.

#### Characterising metric

For analysing the performance of a vaccination strategy, we first simulate a disease spreading on the selected contact networks without vaccination and obtain the outbreak sizes after 35 days. These simulations are run from multiple seed nodes and the average outbreak size *z*_*r*_ is computed. This indicates the propensity of the disease to spread in a network without vaccination and is used as the reference for comparing the effectiveness of the applied vaccination strategies. The performance of a vaccination strategy is quantified by how much reduction it can achieve in terms of average outbreak sizes comparing to *z*_*r*_. Thus, the effectiveness of a vaccination strategy with a vaccination rate *P* is given by

$$\begin{array}{c} \eta =\frac{z_{r}-z_{c|P}}{z_{r}}\times 100 \end{array}$$

where *z*_*c*|*P*_ is the average outbreak sizes of the candidate vaccination strategy with the vaccination rate *P*. In the simulation, the outbreak size indicates the number of new infections caused after running the simulation over 35 days.

## Results

### Pre-emptive vaccination

In this section, the effectiveness of the selected vaccination strategies to prevent future outbreaks in the empirical SPDT contact network (DDT network) and the generated synthetic SPDT contact network (GDT network) are investigated. First, simulations are conducted to understand the upper bound of the efficiency of the applied strategies where it is assumed that contact information of all nodes are available and can be collected for vaccinations. Then, the performance is investigated with the simulation scenarios where contact information of only a proportion of nodes are available for ranking and only these nodes are chosen to be vaccinated. However, all nodes participate in propagating the disease in the networks.

The performance of vaccination strategies is studied with varying vaccination rates *P* (percentage of nodes). Thus, *PN*/100 nodes are chosen based on the vaccination strategies and are vaccinated by assigning their status as recovered. Then, a random node is chosen as a seed node and the outbreak size is obtained by running the disease spreading simulation over 35 days from the seed node. This process is iterated through 5,000 different seed nodes and outbreak sizes are obtained. Seed nodes are infectious for 5 days and then recover. When individuals are asked about their movements, they usually provide information based on the number of individuals they have seen in particular locations. They will not be aware of the number of individuals who have SPDT links through indirect interactions. Thus, the nodes are ranked based on the contacts they have seen, i.e. considering only the direct interactions where both the host and acquaintance nodes stay together in a location. Then, the performance is also examined accounting for the indirect links along with the direct links.

**Fig 1:**
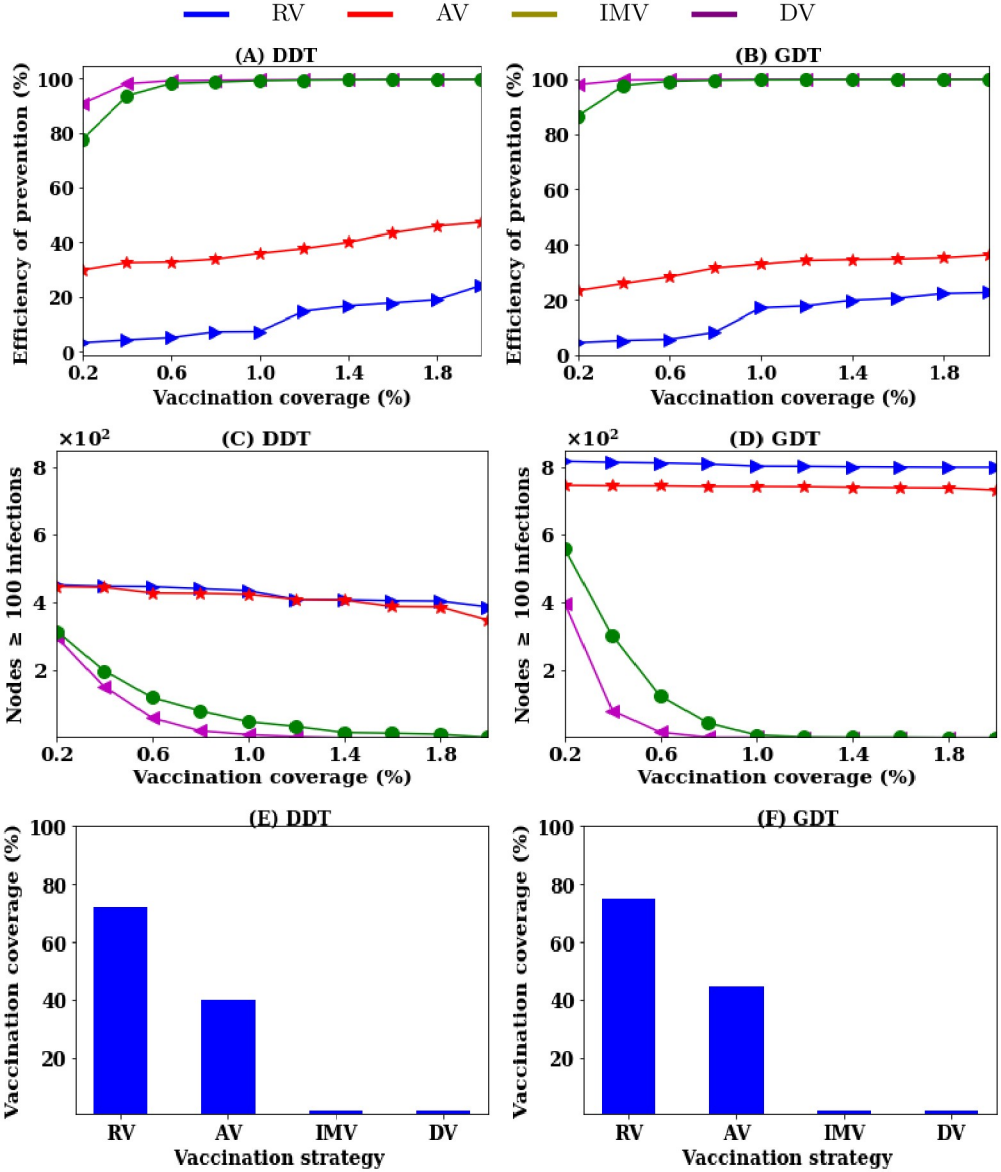
Efficiency of vaccination strategies to prevent disease spreading: (A, B) pre-emptive efficiency as a measure of reduction in average outbreak sizes due to vaccination, (C, D) number of seed nodes out of 5000 having outbreak sizes greater than 100 infections, and (E, F) required vaccination coverage for strategies to keep outbreak sizes below 100 infections from all seed nodes.

By first simulating the disease from 5,000 single seed nodes in both networks, the average outbreak sizes without vaccination are 653 infections in the DDT network and 976 infections in the GDT network of 338K nodes. Based on these values, the pre-emptive efficiency is measured at each *P* for all vaccination strategies and shown in Fig 1(a) and 1(b) while average outbreaks are presented in the Appendix. To clearly understand the efficiency of a strategy, the number of seed nodes having triggered outbreak sizes of more than 100 infections are also counted and presented in Fig 1(c) and 1(d). This indicates the efficiency of a strategy to hinder super-spreader nodes from spreading disease. If there is no seed node with outbreak of 100 infections in a network, it is assumed that the protection from infection is significant for the applied strategy.

The average outbreak sizes quickly decrease when increasing *P* with the IMV strategy. If the vaccination rate is *P* = 0.6% (with vaccinating 2880 nodes), the average outbreak size decreases below 10 infections in both networks and a pre-emptive efficiency of 98% is achieved. However, 1.5% seed nodes have outbreaks of greater than 100 infections and it reaches zero at *P* = 2% vaccination (see Fig 1(C) and 1(D)). On the other hand, RV strategy requires 75% of nodes to be vaccinated to achieve such pre-emptive efficiency in both the DDT and GDT networks (see Fig 1(E) and 1(F)). The AV strategy improves the pre-emptive efficiency over the RV strategy. The AV strategy requires about 40% of nodes to be vaccinated to achieve pre-emptive efficiency where no seed node has outbreaks greater than 100 infections. The DV strategy has the pre-emptive efficiency of 92% in the DDT network and 98% in the GDT network at low vaccination rate. This is slightly higher than the IMV strategy. However, the efficiency of IMV strategy becomes closer to that of DV strategy as *P* increases. To achieve strong pre-emptive efficiency, both strategies require almost the same rates of vaccination. Thus, the coarse-grained information based IMV strategy achieves a similar performance to the degree-based vaccination strategy at a reduced information collection cost.

**Fig 2:**
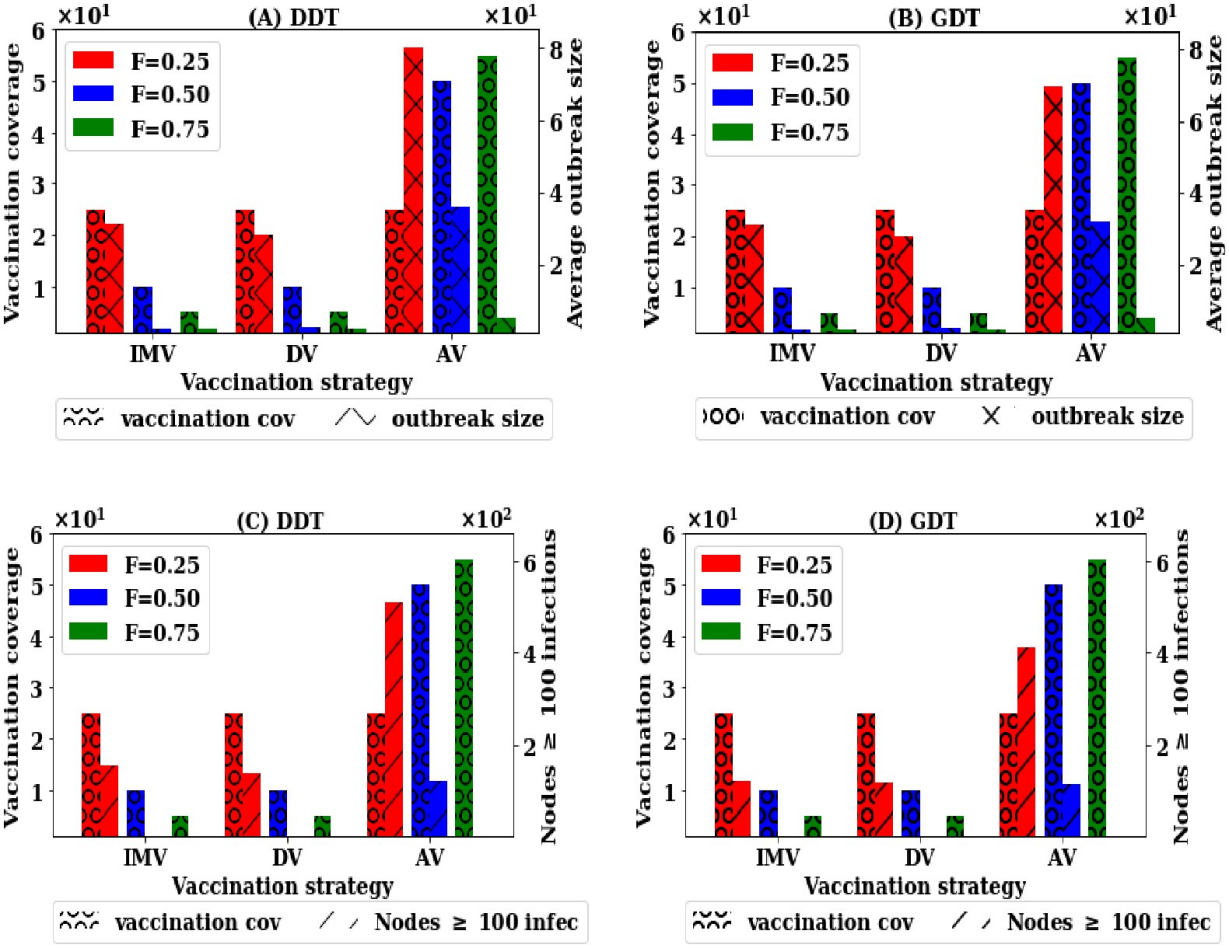
Trade-off among information availability, vaccination coverage and outbreak sizes for various strategies—Vaccination coverage is on the left y-axis while others are on the right y-axis: (A, B) average outbreak sizes with vaccination coverage for different *F*, and (C, D) number of seed nodes having outbreak greater than 100 infections with *P* and *F*.

Now experiments are run to analyse the efficiency of the strategies varying the proportion *F* of nodes that are picked for collecting their movement information. The required vaccination rates *P* for a strategy may depend on the value of *F*. In the simulations, a proportion *F* of nodes are picked up randomly from the first seven days of networks and their ranks are calculated based on the applied vaccination strategy. Then, a vaccination rate *P* is implemented with the sampled nodes. The impact of scale of information availability is studied for varying *F* to 0.25, 0.50 and 0.75. The results are presented in Fig 2.

The results show that there is a trade-off among vaccination coverage, average outbreak sizes and information availability for implementing a vaccination strategy. If the contact information of *F* = 0.25 proportion nodes is applied to select the nodes to be vaccinated, there is still substantially high average outbreak sizes in both DDT and GDT networks even at *P* = 25% (Fig 2) for IMV strategy. Besides, 3% of seed nodes have outbreak sizes more than 100 infections in both networks. Similar results are found for the DV strategy. If *F* is increased to *F* = 0.50, the pre-emptive efficiency increases to 97% in GDT network for IMV strategy and 95% in DDT network at *P* = 10%. However, the degree based vaccination (DV) shows high pre-emptive efficiency compared to that of IMV strategy with lower *P*. The pre-emptive efficiency with no seed node having outbreak sizes greater than 100 infections is achieved by vaccinating 10% of nodes in both networks. At *F* = 0.5, the strong protection from infection can be achieved with a vaccination coverage of 10%. For AV strategy, average outbreak sizes is high up to *F* = 0.5 and there is a large number of seed nodes having outbreak sizes greater than 100 infections. However, the strong pre-emptive efficiency is achievable at *F* = 0.75 with vaccinating of 55% nodes where no seed node has outbreak greater than 100 infections. As RV requires 75% of nodes require to be vaccinated, this is not included in this experiment. With the low scale of information, the IMV strategy performs better than AV strategy and close to DV strategy.

### Reactive vaccination

#### Population level vaccination

**Fig 3:**
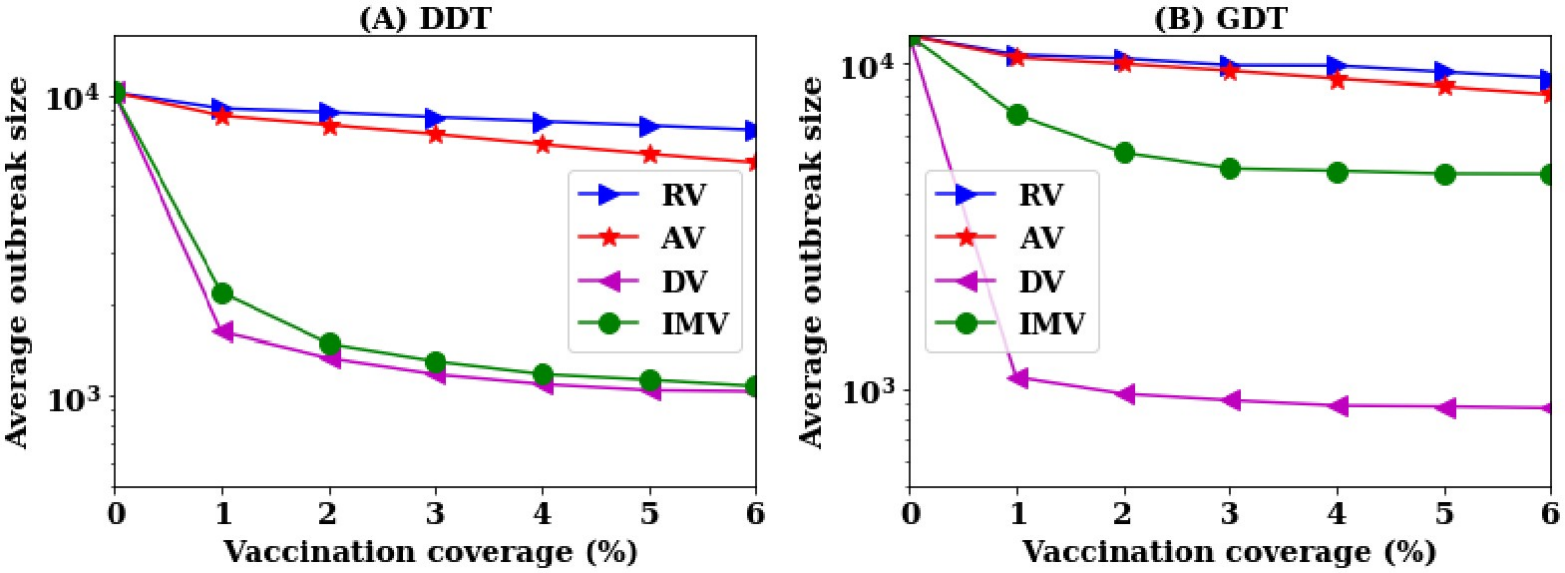
Average outbreak sizes for different vaccination strategies at various vaccination coverage *P* in reactive vaccination.

In these simulations, disease starts with 500 seed nodes and continues for 42 days. Nodes are infectious for *τ* days randomly chosen in the range [3-5] days. The vaccination is implemented at the 7th day of simulation assuming that these days are required to notice the emergence of disease and to collect the contact information. The node’s rank, based on the applied vaccination strategy, is calculated from the contact data of the first seven days. Then, a percentage *P* of nodes are vaccinated (assigned recovered status) and final outbreak sizes are calculated for 42 days of simulations. For each vaccination strategy, simulations are conducted for different *P* in the range [1, 6]% with a step of 1%. At each value of *P*, the simulations are run for 1,000 times and the average outbreak sizes are presented in Fig 3.

The simulation of disease spreading without vaccination creates an outbreak of an average of 10K infections in the DDT network and 12K infections in the GDT network. Random vaccination (RV) and acquaintance vaccination (AV) strategies do not reduce the average outbreak sizes significantly in both networks (Fig 3) without high rates of vaccination. Analysis shows that the RV strategy requires 75% of nodes to be vaccinated in both networks and that the AV strategy requires vaccination of 40% nodes in DDT network and 35% in GDT network to contain disease spreading within the outbreaks of 1000 infections i.e to reduce outbreak sizes by 90%. The proposed vaccination strategy (IMV) shows 90% reduction in outbreak sizes with vaccinating 4% of nodes. The degree-based vaccination shows similar performance to that of the IMV strategy. Similar to the pre-emptive vaccination, the DV strategy initially shows better performance and then becomes similar to the IMV strategy at the higher vaccination rates. The coarse-grained information based IMV strategy achieves the same performance of DV strategy for the reactive vaccination scenarios as well.

**Fig 4:**
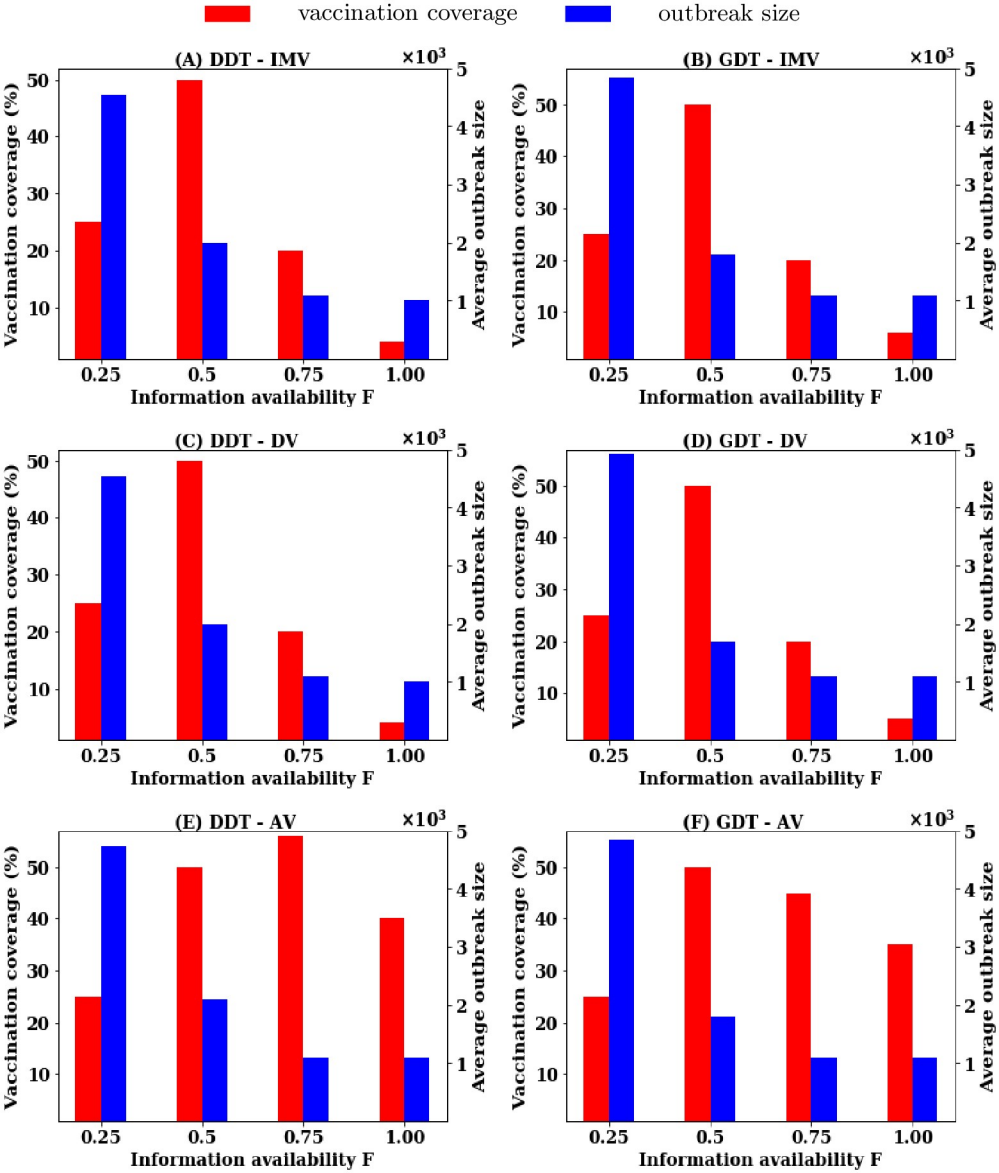
Trade-off among information availability, vaccination coverage and average outbreak sizes in population level vaccination: (A, B) proposed IMV strategy, (C, D) degree based strategy (DV), and (E,F) acquaintance vaccination (AV) strategy.

Simulations are now conducted to understand the effectiveness of vaccination strategies with the scale of information availability regarding node’s contact for reactive scenarios. Simulations are carried out for the scenarios where the contact information of *F* = {0.25, 0.5, 0.75} proportion of nodes are available for ranking procedure. The simulations are also run for all strategies until the average outbreak sizes are reduced by 90% (1000 infections) at a certain value of *P*. The results obtained show the trade-off among information availability, vaccination coverage and average outbreak sizes for strategies and are presented in Fig 4.

Results show that no strategy does reduce outbreak size below 6000 infections at *F* = 0.25 in both DDT and GDT networks for any value of *P*. By increasing *F* to 0.50, both IMV and DV strategies are capable of reducing the outbreak sizes by 80% with vaccinating all nodes picked up for ranking procedure. This is also the same with the AV strategy (Fig 4). Increasing *F* to 75% reduces the requirement of vaccination rate significantly with both IMV and DV. IMV strategy requires 25% of the nodes to be vaccinated and DV strategy requires 20% in both networks. At this value of *F*, acquaintance vaccination (AV) shows the ability to contain outbreak sizes below 1000 infections (outbreak sizes reduced by 90%) with 55% vaccination in the DDT network and 45% vaccination in the GDT network. There is also a strong trade-off between average outbreak sizes, vaccination coverage and average outbreak sizes in applying a vaccination strategy (Fig 4) in population-level reactive vaccination scenario.

#### Node level vaccination

**Fig 5:**
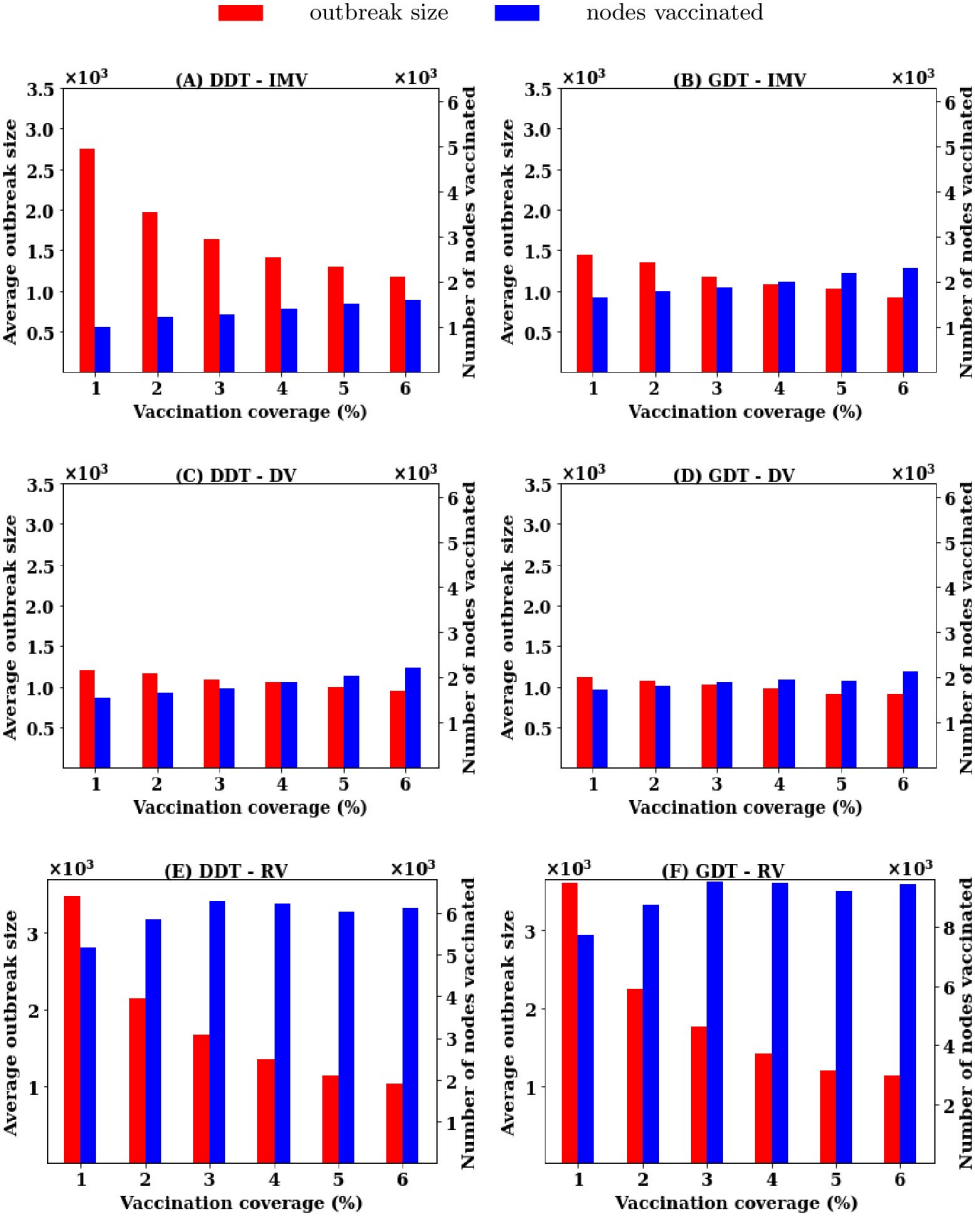
Performance of node level vaccination with various strategies: (A, B) proposed IMV strategy, (C, D) DV strategy, and (E,F) RV strategy.

In node level vaccination, a percentage of an infected node’s neighbours is vaccinated. In this experiment, neighbour nodes to be vaccinated are selected using three methods, namely randomly, degree-based ranks and IMV based ranks. Similar to the previous experiments, the disease starts with 500 seed nodes and continues for 42 days. Initially, nodes are vaccinated at the 7th day of simulation and after that neighbour nodes are vaccinated when a new node is infected. It is assumed in the first experiment that all infected nodes are identified and their neighbour nodes are vaccinated based on applied strategy. Then, a proportion *F* of all infected nodes is identified and their neighbour nodes are vaccinated. In these simulations, a threshold value, C, of the ranking score is set to select neighbour nodes following DV and IMV strategies. The value of C is a ranking score above which a percentage *P* of total nodes have scored. If a neighbour node has scored more than C, it will be vaccinated. In random vaccination (RV), a percentage *P* of neighbour nodes are randomly chosen for vaccination. The results are presented in Fig 5.

Node level vaccination is more efficient than the population level vaccination. In IMV strategy, the outbreak sizes are reduced by 90% in both networks for C corresponding to *P* = 6% with the vaccination of 0.6% (2,000) nodes. To achieve this performance in population level vaccination, IMV strategy requires vaccination of about 4% (14,400) nodes. The DV strategy reduces average outbreak size by 90% for C corresponding to *P* = 4% with the vaccination of 0.6% (2,000) nodes. However, RV strategy cannot reduce average outbreak sizes significantly until more than 30% of neighbour nodes are vaccinated. It is also found that the number of nodes vaccinated is stabilised at about 6,000 nodes in the DDT network and 10,000 nodes in the GDT network regardless of *P* in RV strategy. For node level vaccination, coarse-grained information based IMV strategy achieves the performance of DV strategy and better than RV strategy.

**Fig 6:**
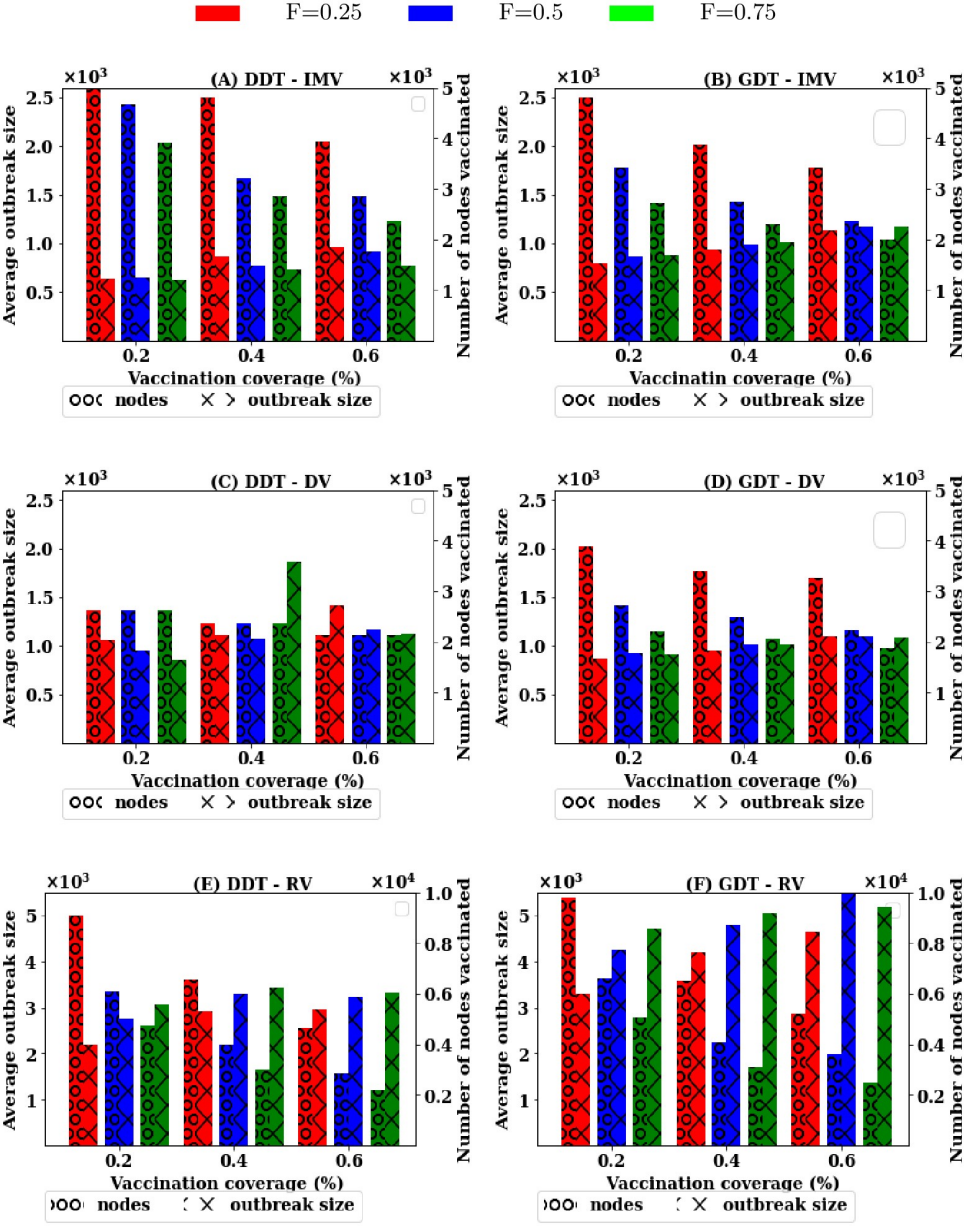
Performances of node level vaccination with F proportion of infected nodes identified: (A, B) proposed vaccination strategy, (C, D) degree based vaccination, and (E, F) acquaintance based vaccination.

**Fig 7:**
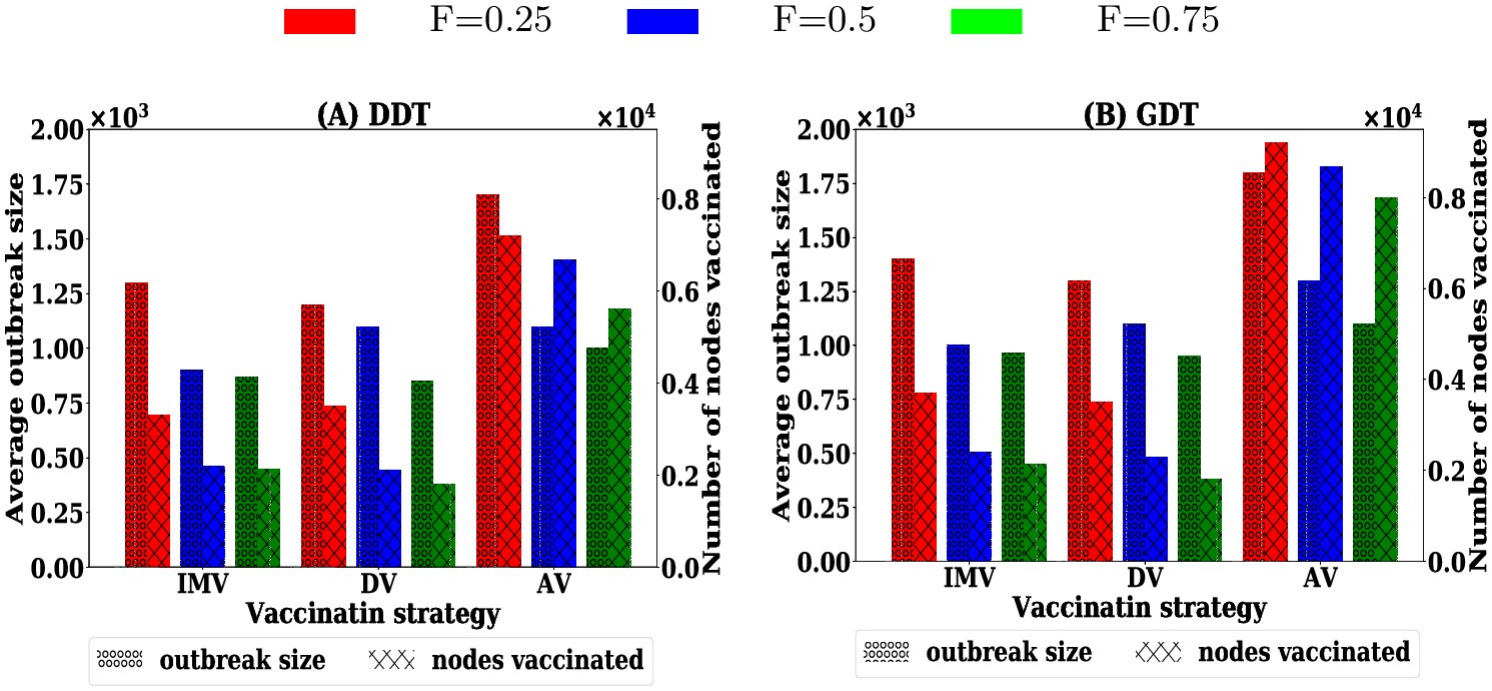
Required number nodes to be vaccinated in node level vaccination for achieving outbreak sizes below 1000 infections with F proportion of infected nodes identified with different strategies.

The performance is now studied varying *F*, the proportion of infected nodes can be identified, for each vaccination strategy. In these simulations, a proportion *F* of infected nodes are selected and then the threshold based procedures of vaccinating nodes are used according to the applied vaccination strategy. The average outbreak sizes and the corresponding number of nodes vaccinated at different *F* for each vaccination strategy are presented in Fig 6. At *F* = 0.25, no strategies can reduce the outbreak sizes below 90% in both networks. At *F* = 0.5, IMV and RV strategies still cannot reduce the average outbreak sizes by 90% with any value of *P*, but DV strategy can do by vaccinating 4,000 nodes. Every strategy can reduce the average outbreak sizes by 90% while IMV and DV require vaccination of 2000 nodes and RV requires vaccination of 6,000 nodes(Fig 7).

## Discussion

The IMV strategy achieves a pre-emptive vaccination efficiency similar to the DV strategy and significantly better than both AV and RV strategies. IMV strategy under-performs compare to DV strategy at low vaccination rates, but it becomes similar to DV when higher preventive efficiency is required. With the constraint of contact information collection, IMV and DV strategies can prevent disease spread if we know the contact information of more than 50% nodes with vaccinating 10% of nodes. However, DV strategy depends on the exact contact information which is not always possible to collect or being remembered by individuals. Thus, a coarse-grained contact information based method such as IMV is more appropriate for real-world vaccinations.

Considering reactive vaccinations, IMV strategy achieves a similar performance compared to DV strategy. IMV can reduce outbreak sizes by 90% with vaccination of 4% nodes in population approach. However, the scale of collecting contact information severely impact the reactive vaccination performance. No vaccination method achieve good efficiency under 50% contact information. IMV and DV strategies require about 10% of nodes to be vaccinated at F = 0.75. Similar performances of IMV are observed when it is applied for node level reactive vaccination. In all experiments, GDT networks show higher outbreak sizes. This is because nodes in the GDT network contact more nodes and also have more frequent contacts than with DDT networks. Therefore, more infections are caused by an infected node in the GDT network.

**Fig 8:**
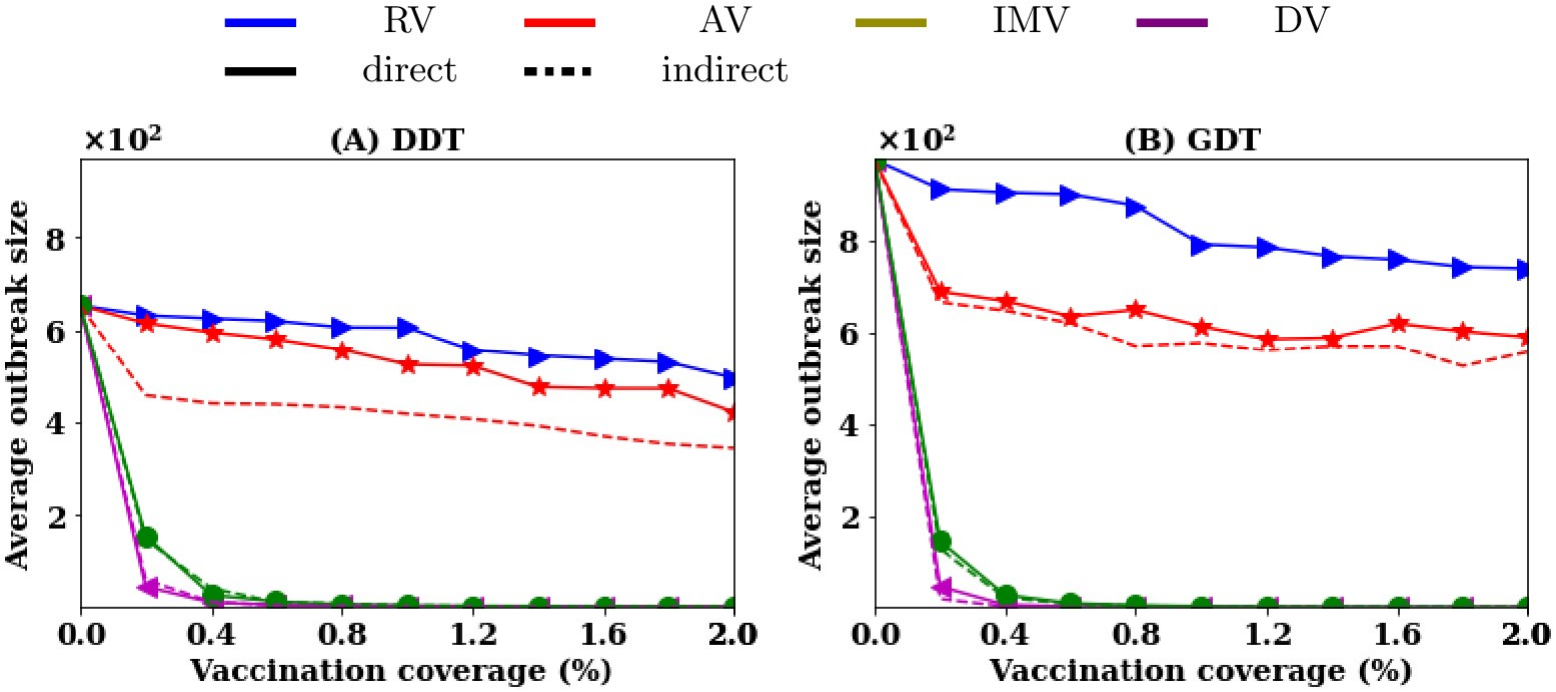
Comparison of outbreak sizes for vaccinating nodes with contacts based on the direct interactions (solid lines) and contact based on any direct or indirect interactions (dashed lines).

The above performances are obtained by ranking the nodes based on the contacts created for direct interactions, i.e. neighbour nodes who are connected with direct links are only considered in the ranking process. Fig 8 shows (dashed lines) the results for vaccination strategies with indirect interactions, i.e. neighbour nodes connected with indirect links are also considered in calculating the ranking scores for a strategy. It is observed that both DV and IMV strategies do not vary the average outbreak sizes largely for any vaccination rates with the inclusion of indirect links. As the movement information is the same for creating direct or indirect interactions, the performance of IMV strategy does not decrease. The performance of the other neighbour based strategy AV increases slightly when considering indirect interactions as some nodes may become important with indirect links and this is not captured by the direct interaction based implementation.

**Fig 9:**
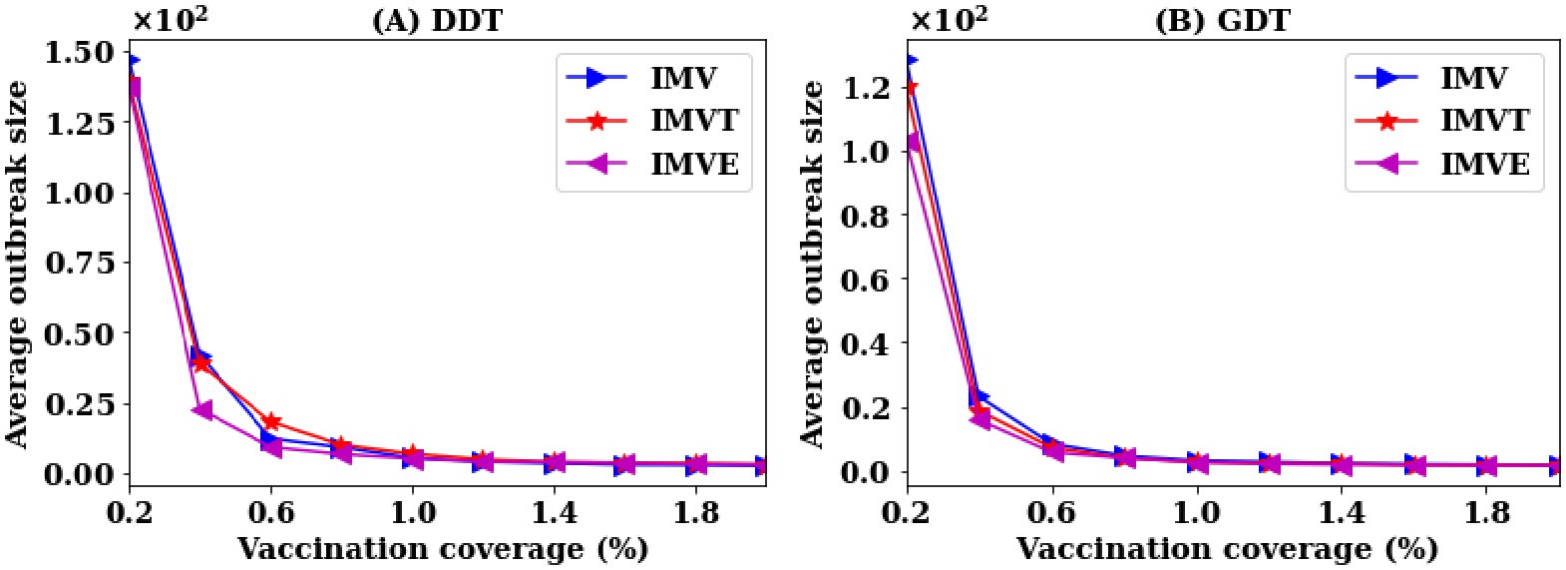
Variations in average outbreak sizes of individual’s movement based vaccination (IMV) strategy accounting for the temporal information and exact contact information. IMVT means IMV strategy with temporal information and IMVE means IMV strategy with exact contact information.

Fig 9 presents how much improvement can be achieved if exact contact information of nodes is applied by ranking process of IMV strategy. Nodes are now ranked with the exact contact information and temporal information. Theses vaccination strategies are named as IMVE (exact information based) and IMVT (with temporal information based). To account temporal dependency, the transmission probability *β* is defined as

$$\begin{array}{c} \beta =1.6\beta_{0}(1-e^{-\frac{t_{i}}{t_{0}}}) \end{array}$$ (3)

where *t*_*i*_ is the stay time of the node for a visit *i* to a location, *t*_0_ is the average stay time of users of Momo App, and *β*_0_ is the disease transmission probability if a susceptible node stays with an infected node for *t*_0_ time (see Appendix for details).

Applying exact information, the IMVE strategy shows a slightly lower average outbreak sizes at lower *P*. However, at *P* = 0.8%, the average outbreak sizes close to one for all versions of IMV strategy. Thus, it is concluded that the inclusion of temporal information (stay duration) does not have a substantial impact when the requirement of pre-emptive efficiency is high and a high vaccination rate is applied. The coarse-grained contact information is sufficient to capture the individual movement information.

This study has some limitations that might affect the results. The location classes are assumed intuitively based on the possible number of individuals can be meet if these locations are visited. The DDT networks are built using the location updates of Momo users. However, the contacts of Momo users with other individuals were not counted which might have potential disease transmission links. Thus, there might be under estimate of outbreak sizes. Some of this is adjusted in the GDT network. The infection risk model is based on simplified assumptions that may vary the disease transmission in the real-world scenarios. In the vaccination strategy design, one may need to consider some practical conditions such as limitation of human resources, delay to distribute vaccine, and priorities for some specific community, etc.

There are several interesting directions for future works. We can increase the accuracy of classification investigating locations updates of Momo users and Map technology. The other interesting direction is to study how the strategies are affected based on the disease transmission intensities as different diseases have different infectiousness. Developing mathematical models and formulas for the efficiency of vaccination strategies would helpful for the relevant communities.

## Conclusion

This paper has investigated vaccination strategies on dynamic contact networks having indirect transmission links. We have developed a simple vaccination strategy called individual movement based vaccination (IMV). This strategy can work with the coarse-grained and easily accessible contact information, such as places visited, instead of exact contact information, such as the exact list of individuals possibly contacted. The IMV strategy is examined for both pre-emptive and reactive scenarios. The performance is compared with other three theoretical vaccination strategies commonly used in literature, namely random vaccination (RV), acquaintance vaccination (AV) and degree based vaccination (DV).

Overall, the IMV strategy shows similar performances than the DV strategy and outperform the other two strategies, while not relying on the unrealistic expectation to know each individual contact. All vaccination strategies are also studied against the scale of information availability on all nodes. Interestingly it is found that all strategies have the maximum performance of RV strategy if 50% of nodes provide contact information for the vaccination procedure. Finally, it is important to note that this theoretical study considered both direct and indirect interactions, which re-enforces the interest of the IMV strategy as it has the same performance for both cases.

## A Appendix

### A.1 Contact networks generation

This section explains the construction of contact network from the selected Momo data set. All possible disease transmission links are extracted according to the SPDT diffusion model [17, 18]. In the SPDT model, a link is created if a susceptible individual visits a location where an infected individual has been to that location within a time frame. As the first step of contact network construction, it is identified that an host user (assumed infected with disease) *v* is staying at a location. Consecutive updates, *X* = {(*x*_1_, *t*_1_), (*x*_2_, *t*_2_), …(*x*_*k*_, *t*_*k*_)} where *x*_*i*_ are the coordinate values and *t*_*i*_ are the update times, from the user *v* within a radius of 20m (travel distance of airborne infection particles [30]) of the initial update at location *x*_1_ are indicative of the user staying within the same proximity of *x*_1_. For the host user *v*, its visit to the proximity of *x*_1_ will represent an active visit if a neighbouring user *u* has location updates starting at $t_{1}^{{}^{\prime }}$ while *v* is present, or within *δ* seconds after *v* leaves the location. The user *u* should have at least two updates within 20m of *x*_1_ to ensure that it is in fact staying at the same proximity, and therefore can be exposed to the infected particles, rather than simply passing by. The stay period of host user *v* at the proximity of *x*_1_ is (*t*_*s*_ = *t*_1_, *t*_*l*_ = *t*_*k*_), where *t*_*k*_ represents the end of the current stay period. If *u*’s last update within 20m around *x*_1_ is $\left(x_{j}^{{}^{\prime }},t_{j}^{{}^{\prime }}\right)$, the created SPDT link has a link duration ($t_{s}^{\prime }=t_{1}^{\prime },t_{l}^{\prime }=t_{j}^{{}^{\prime }}$) due to active visit (*t*_*s*_ = *t*_1_, *t*_*l*_ = *t*_*k*_). All links to other users for this active visit (*t*_*s*_ = *t*_1_, *t*_*l*_ = *t*_*k*_) are computed. Similarly, all visits made by *v* are searched within the updates over the 32 days and relevant SPDT links are extracted. Each link between the two same users are distinguished by the time intervals (*t*_*s*_, *t*_*l*_) and $\left(t_{s}^{\prime },t_{l}^{\prime }\right)$.

### A.2 Disease transmission probability

The disease transmission probability are calculated for both the direct and indirect contacts as follows. If a node in the susceptible compartment receives a SPDT link from a node in the infectious compartment, the former is subject to exposure *E*_*l*_ of infectious pathogens for both direct and indirect transmission links according to the following equation

$$\begin{array}{c} E_{l}=\frac{gp}{Vr^{2}}[r(t_{i}-t_{s}^{\prime })+e^{rt_{l}}(e^{-rt_{i}}-e^{-rt_{l}^{\prime }})]+\frac{gp}{Vr^{2}}(e^{-rt_{l}^{\prime }}-e^{-rt_{s}^{\prime }})e^{rt_{s}} \end{array}$$ (4)

where g is the particle generation rate of infected individual, p is the pulmonary rate of susceptible individual, V is the volume of the interaction area, r is the particles removal rates from the interaction area, *t*_*s*_ is the arrival time of the infected individual, *t*_*l*_ is the leaving time of the infected individual, $t_{s}^{\prime }$ is the arrival time of susceptible individuals and $t_{l}^{\prime }$ is the leaving time of susceptible individuals from the interaction location and *t*_*i*_ is given as follows: $t_{i}=t_{l}^{\prime }$ when the SPDT link has only a direct component, *t*_*i*_ = *t*_*l*_ if the SPDT link has both direct and indirect components, and $t_{i}=t_{s}^{\prime }$ otherwise. If $t_{s}<t_{s}^{\prime }$, *t*_*s*_ is set to $t_{s}^{\prime }$ for calculating an appropriate exposure [17]. If a susceptible individual receives *m* SPDT links from infected individuals during an observation period, the total exposure *E* is

$$\begin{array}{c} E=\sum_{k=0}^{m}E_{l}^{k} \end{array}$$ (5)

where $E_{l}^{k}$ is the received exposure for k^th^ link. The probability of infection for causing disease can be determined by the dose-response relationship defined as

$$\begin{array}{c} P_{I}=1-e^{-\sigma E} \end{array}$$ (6)

where *σ* is the infectiousness of the virus that causes infection [29].

### A.3 Sensitivity analysis

To analyse the sensitivity, it is required to add exact contact information and stay time for a location. Thus, a new ranking score is defined as

$$\begin{array}{c} w_{i}=1-{\left(1-\beta \right)}^{d_{i}} \end{array}$$ (7)

where *w*_*i*_ is the probability of transmitting disease to neighbouring nodes for a visit *i* at a location where infected nodes meet *d*_*i*_ number of other nodes. Thus, the node’s rank can be given by

$$\begin{array}{c} W=\sum_{i=1}^{n}w_{i} \end{array}$$ (8)

This equation now finds the score considering the exact number of neighbouring nodes for each visit during the observation period. The other important factor for disease spread through visiting the location is the duration of stay. If an infected individual stays longer at a location, they may transmit disease to more susceptible individuals. Thus, the temporal information is integrated with the ranking process as follows. It is assumed that the probability of transmitting disease for visiting a location increases exponentially. Therefore, the transmission probability *β* is defined as

$$\begin{array}{c} \beta =1.6\beta_{0}(1-e^{-\frac{t_{i}}{t_{0}}}) \end{array}$$ (9)

where *t*_*i*_ is the stay time of the node for a visit *i* to a location, *t*_0_ is the average stay time of users of Momo App, and *β*_0_ is the disease transmission probability if a susceptible node stays with an infected node for *t*_0_ time. Integrating this time dependency on transmission probability of Eq 7 can capture the impact of the duration of stay at the visited locations. Therefore, the IMV strategy is also studied based the node’s rank with temporal information. Finally, the performance of all vaccination strategies is studied for the scenarios where *F* proportion of nodes provides contact information for the ranking process and nodes to be vaccinated are chosen from them. The experiment is conducted for *F* = {0.25, 0.5, 0.75}.

### A.4 Outbreak sizes for pre-emptive vaccination

**Fig 10:**
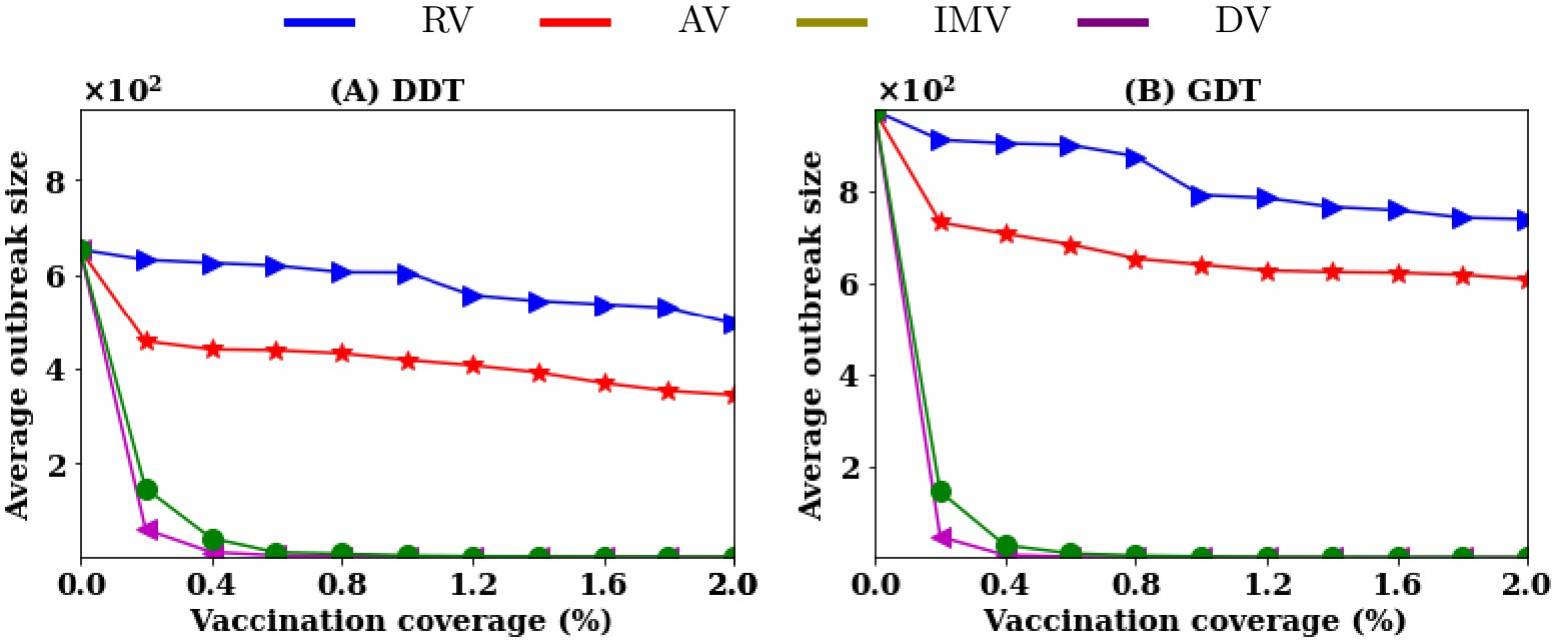
Average outbreak sizes for 5,000 simulations at various vaccination coverage *P* (percentage of total nodes) of different strategies.

The performances of vaccination strategies for pre-emptive vaccination is studied with varying vaccination rates *P* (percentage of nodes) in the range [0.2-2]% with the step of 0.2%. The results are presented in Fig 10.

### A.5 Impact of scale of information availability for pre-emptive vaccination

**Fig 11:**
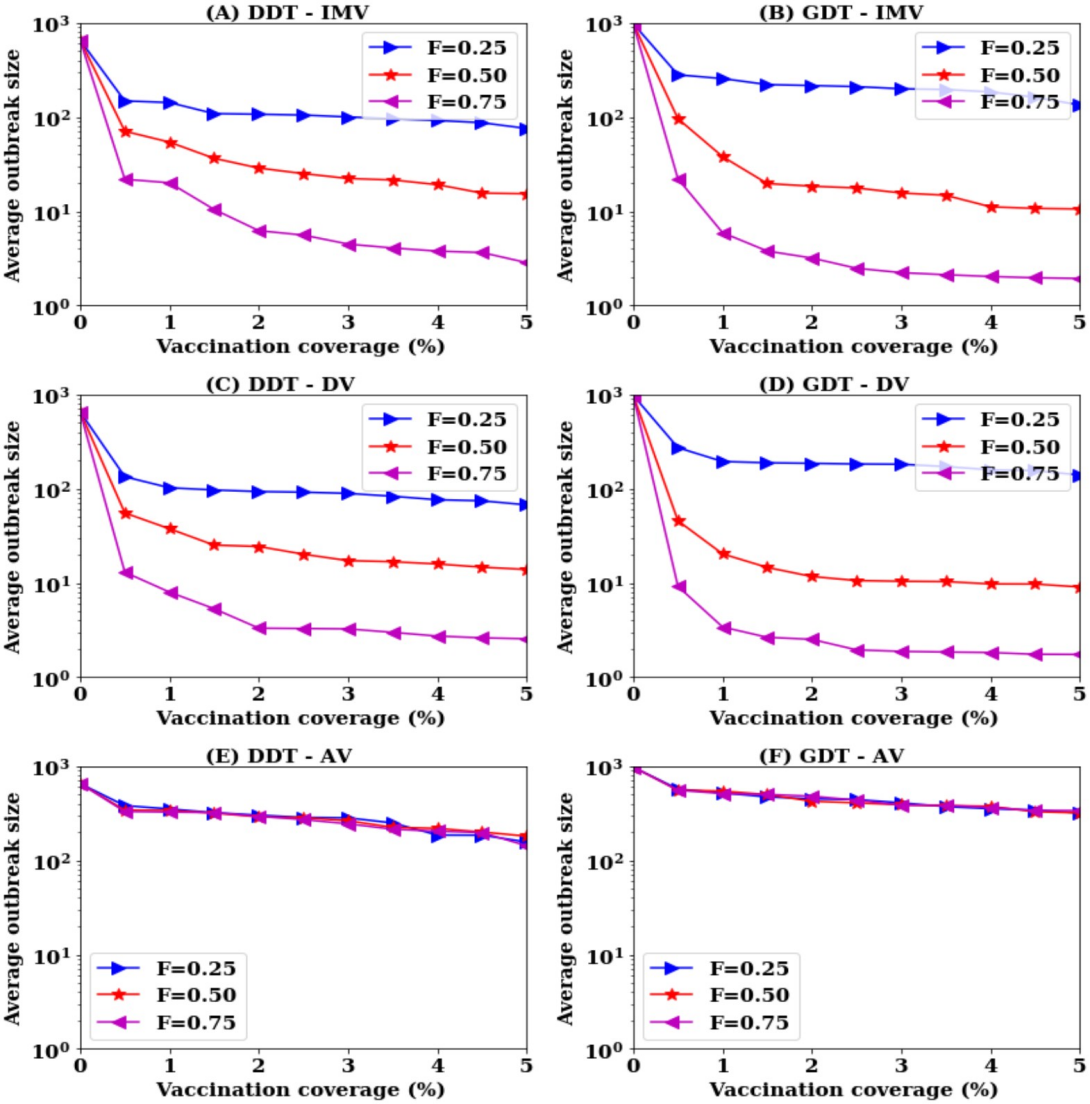
Performance of vaccination strategies at various scales of information availability *F*: (A,B) proposed vaccination strategy, (C,D) degree based vaccination strategy and (E,F) acquaintance vaccination strategy.

The impact of scale of information availability is studied for varying *F* to 0.25, 0.50 and 0.75. The simulations are conducted for IMV, DV and AV strategies for pre-emptive scenario on both DDT and GDT networks. The average outbreak sizes are presented in Fig 11.

### A.6 Impact of scale of information availability for reactive vaccination

**Fig 12:**
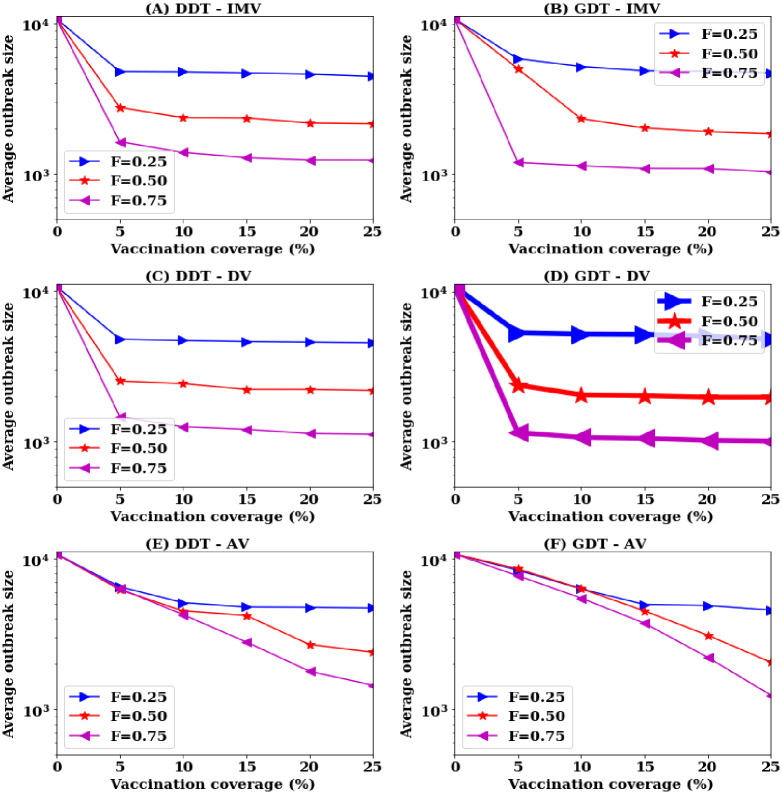
Variation in reactive performances with scale of information availability of node contacts: (A,B) proposed vaccination strategy, (C,D) degree based vaccination, and (E,F) acquaintance based vaccination.

Simulations are carried out for the scenarios where the contact information of *F* = {0.25, 0.5, 0.75} proportion of nodes are available for ranking procedure in reactive population level vaccination. At each value of *F*, the performance of the vaccination strategies is analysed for vaccination rates *P* varying in the range [0, 25]% with a step of 5%. The results are presented in Fig 12.
